# Hepatic stellate cells in zone 1 engage in capillarization rather than myofibroblast formation in murine liver fibrosis

**DOI:** 10.1038/s41598-024-69898-z

**Published:** 2024-08-13

**Authors:** Muhammad Ashfaq Khan, Julian Fischer, Leon Harrer, Fabian Schwiering, Dieter Groneberg, Andreas Friebe

**Affiliations:** Physiologisches Institut, Julius-Maximilians-Universität Würzburg, 97070 Würzburg, Germany

**Keywords:** Lineage tracing, Pericyte, Smooth muscle myosin heavy chain, Liver lobule, Transgenic mouse, Physiology, Diseases, Gastroenterology, Medical research

## Abstract

The combination of lineage tracing and immunohistochemistry has helped to identify subpopulations and fate of hepatic stellate cells (HSC) in murine liver. HSC are sinusoidal pericytes that act as myofibroblast precursors after liver injury. Single cell RNA sequencing approaches have recently helped to differentiate central and portal HSC. A specific Cre line to lineage trace portal HSC has not yet been described. We used three Cre lines (Lrat-Cre, PDGFRβ-CreER^T2^ and SMMHC-CreER^T2^) known to label mesenchymal cells including HSC in combination with a tdTomato-expressing reporter. All three Cre lines labeled populations of HSC as well as smooth muscle cells (SMC). Using the SMMHC-CreER^T2^, we identified a subtype of HSC in the periportal area of the hepatic lobule (termed zone 1-HSC). We lineage traced tdTomato-expressing zone 1-HSC over 1 year, described fibrotic behavior in two fibrosis models and investigated their possible role during fibrosis. This HSC subtype resides in zone 1 under healthy conditions; however, zonation is disrupted in preclinical models of liver fibrosis (CCl_4_ and MASH). Zone 1-HSC do not transform into αSMA-expressing myofibroblasts. Rather, they participate in sinusoidal capillarization. We describe a novel subtype of HSC restricted to zone 1 under physiological conditions and its possible function after liver injury. In contrast to the accepted notion, this HSC subtype does not transform into αSMA-positive myofibroblasts; rather, zone 1-HSC adopt properties of capillary pericytes, thereby participating in sinusoidal capillarization.

## Introduction

Hepatic stellate cells (HSC) have been considered a homogenous hepatic mesenchymal cell population that comprises about 5–8% of total liver cells, resides in the space of Disse and has important roles in normal liver physiology and fibrogenesis^1^. Under physiological conditions, HSC are quiescent and store vitamin A. After injury, HSC are activated and then act as myofibroblast precursors to produce extracellular matrix, matrix-degrading metalloproteinases and proinflammatory/profibrogenic cytokines^2,3^. in fact, 82–96% of myofibroblasts were shown to derive from HSC under fibrotic conditions^4,5^. In fibrotic liver tissue, myofibroblasts can be identified by the de novo expression of the contractile fiber α smooth muscle actin (αSMA), a classical marker of HSC activation^6,7^.

Despite numerous studies on the role of HSC during liver fibrosis, the heterogeneity of HSC under physiological conditions has come into focus only recently. Single cell RNA sequencing-based studies have started to reveal the physiological and pathophysiological heterogeneity of HSC based on spatial and functional aspects^8^. In a seminal study, Dobie et al. have broadly grouped HSC into two subtypes, namely central vein-associated HSC (CaHSC) and portal vein-associated HSC (PaHSC)^9^. Spatial zonation of these two HSC subtypes was demonstrated by the combination of differential gene cluster expression and immunohistology: NGFR (nerve growth factor receptor) was shown to be highly expressed in PaHSC whereas ADAMTSL2 (an extracellular matrix glycoprotein) was more prominent in CaHSC. Whereas CaHSC were shown to turn into collagen-producing myofibroblasts in a mouse model of centrilobular fibrosis, the function of PaHSC remained open.

In a Tcf21 (transcription factor 21)-based lineage tracing approach, Wang et al. were able to label approx. 10% of the HSC, which were mainly located at periportal and pericentral zones under physiological conditions^10^. Using CCl_4_, perivenous stellate cells were shown as main source of myofibroblasts whereas the fate of periportal HSC was not further elucidated in this fibrosis model. Another single cell RNA-seq study^8^ also showed two subpopulations of HSC based on differential gene expression and spatial zonation: in slight contrast to the HSC localization described by Dobie et al., this study differentiates portal and central vein-concentrated HSC (positive for GPC3, a cell surface proteoglycan) and perisinusoidally located HSC (positive for dopamine beta hydroxylase; DBH). Functionally, both subpopulations participate in ECM production but differ in their functions such as the expression of genes involved in glycosaminoglycan metabolism and antigen presentation. Taken together, these studies report zone- and/or function-specific subtypes of HSC, but their identification appears to be highly dependent on the marker(s) employed.

Using a lineage tracing approach with three different Cre-reporter lines (Lrat-Cre, PDGFRβ-CreER^T2^ and SMMHC-CreER^T2^) in combination with a tdTomato-expressing reporter, we were able to label all HSC with the Lrat-Cre and subsets of HSC with the other two Cre lines. All three Cre lines labeled hepatic SMC. With the SMMHC-CreER^T2^/tdTomato mouse line, we here identify a subtype of HSC that localizes in zone 1 of the hepatic lobule (termed zone 1-HSC). In the CCl_4_ model of hepatic fibrosis, zone 1-HSC lose their zonation and migrate into central zones. Zone 1-HSC do not transform into αSMA/collagen 1-expressing myofibroblasts in two different fibrosis models (CCl_4_ and Western diet). Our data indicate that zone 1-HSC participate in the sinusoidal capillarization after CCl_4_ injury. In summary, we here describe the novel function of an HSC subtype which is specifically found in zone 1 of the healthy liver.

## Results

### Identification of a subtype of stellate cells in the murine liver

We first compared three Cre lines known to target HSC/pericytes as well as SMC (Supplementary Fig. 1). Reporter mice expressing the fluorescent dye tdTomato under control of the PDGFRβ, Lrat or SMMHC promoter were obtained by crossing PDGFRβ-CreER^T2^, Lrat-Cre and SMMHC-CreER^T2^ with a tdTomato reporter line. The Lrat-Cre tomato-labeled all HSC whereas stochastic labeling of HSC was seen under control of the PDGFRβ-CreER^T2^. Induction of SMMHC-CreER^T2^ led to tomato-labeling of HSC in the periportal region of the hepatic lobule.

**Figure 1:**
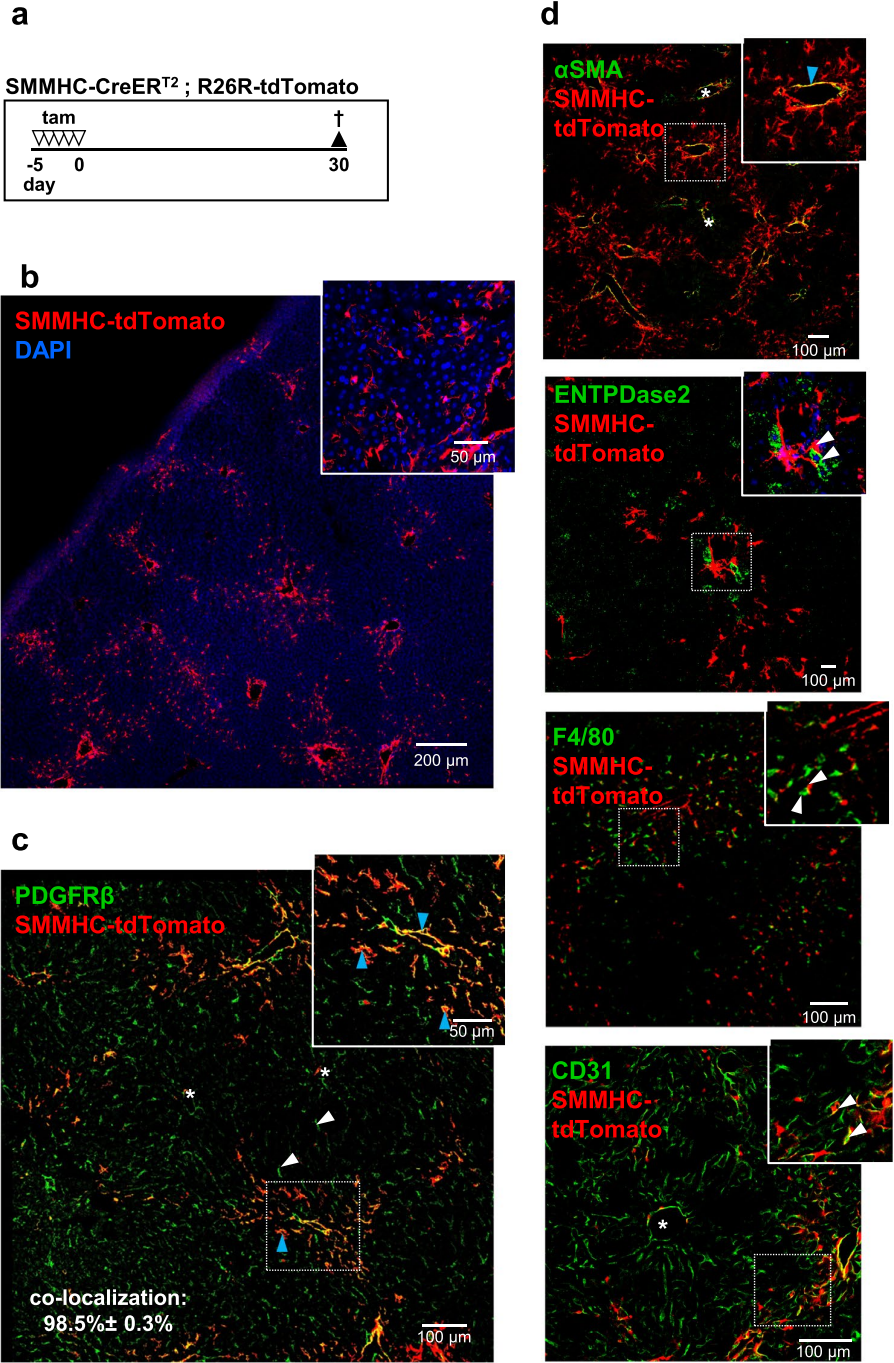
Identification of SMMHC-CreER^T2^-labeled stellate cells in the murine liver. (**a**) SMMHC-tdTomato reporter mice were injected with tamoxifen (tam) on 5 consecutive days to induce the expression of the fluorescent dye tdTomato under control of the SMMHC promoter (SMMHC-tdTomato). 30 days later, mice were sacrificed, and livers were then isolated, fixed and analyzed. (**b**) tdTomato fluorescence in the liver (red). DAPI staining (blue) indicates nuclei. High resolution image shows stellate shape of tdTomato-positive cells. (**c**) SMMHC-tdTomato-positive HSC are stained with an antibody against PDGFRβ (green); quantification indicated 98.5% ± 0.3% of the tdTomato-expressing cells to be positive for PDGFRβ (cells were counted from n = 2 images (20 × magnification) from N = 3 animals; mean ± SEM). (**d**) SMMHC-CreER^T2^-induced tdTomato was also found in smooth muscle cells (antibody against αSMA; green), but neither in portal fibroblasts, endothelial cells nor Kupffer cells as shown with antibodies against ENTPDase2, CD31 and F4/80 (all green). Asterisks in c and d indicate central veins. Enlargements in c and are indicated by dotted squares. Blue arrowheads indicate co-expression, white arrowheads indicated lack of co-expression.

As the zonated periportal tdTomato expression indicated a spatially confined HSC subtype, we further characterized this cell type using the SMMHC-CreER^T2^/tdTomato reporter. The SMMHC-CreER^T2^ mouse was originally published as a smooth muscle cell-specific line^11^ but later recognized to also target capillary pericytes in several organs^12,13^^14^. Liver sections from tamoxifen-treated SMMHC-CreER^T2^-tdTomato reporter mice (SMMHC-tdTomato; Fig. 1a, induction scheme) showed discrete fluorescence labelling in the liver parenchyma (Fig. 1b). This staining could be attributed mainly to HSC as staining with an antibody against PDGFRβ, a marker for mesenchymal cells including HSC^4,9,15^; tdTomato-labeled cells appeared to be primarily located in the portal area of the hepatic lobule (Fig. 1c; co-localization of SMMHC-tdTomato with PDGFRβ stain: 98.5% ± 0.3%). As expected for the SMMHC promoter, tdTomato labeling was also detected in SMC of the portal and central areas (αSMA, Fig. 1d). SMMHC-CreER^T2^-induced tdTomato expression was found neither in portal fibroblasts (ENTPDase2), endothelial cells (CD31) nor in Kupffer cells (F4/80) (Fig. 1d). Taken together, in the murine liver, SMMHC-CreER^T2^ is active in SMC and in a subtype of HSC located in the portal area of the hepatic lobule.

### SMMHC-CreER^T2^-labeled HSC are located in zone 1 of the hepatic lobule

**Figure 2:**
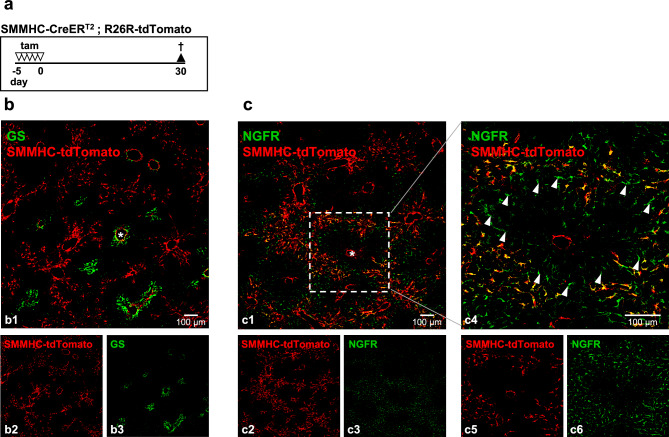
SMMHC-CreER^T2^-labeled HSC are located in zone 1 of the hepatic lobule. (**a**) SMMHC-tdTomato reporter mice were injected with tamoxifen (tam) on 5 consecutive days to induce the expression of the fluorescent dye tdTomato under control of the SMMHC promoter (SMMHC-tdTomato). 30 days later, mice were sacrificed, and livers were then isolated, fixed and analyzed. tdTomato fluorescence is shown in red. (**b**) Antibodies against glutamine synthetase (GS, green) were used to identify hepatocytes around the central vein. (**c**) Antibodies against nerve growth factor receptor (NGFR, green) were used to identify portal vein-associated HSC according to Dobie et al.^9^. The enlargement in c4 shows tdTomato-negative, NGFR-positive cells towards the center of the lobule. Asterisks indicate central veins. Single channels are shown in b2/b3, c2/c3 and c5/c6.

To determine the localization of SMMHC-tdTomato-positive HSC more precisely, we used the zonation markers glutamine synthetase (GS) and nerve growth factor receptor (NGFR) which label hepatocytes around the central vein^16,17^ and portal HSC^9^ respectively (Fig. 2). GS labeling clearly differentiated tdTomato-expressing SMC of the central vein (SMC surrounded by GS-positive cells) from tdTomato-expressing cells distant from the central vein (Fig. 2b). Dobie et al.^9^ established NGFR as marker for 'portal vein-associated HSC' (NGFR^hi^ HSC) which, in combination with 'central vein-associated HSC' (NGFR^low^ HSC) form two different subtypes of stellate cells. NGFR did not label SMC but was co-localized with tdTomato in most but not all HSC in zone 1 (Fig. 2c). In fact, NGFR-positive but tdTomato-negative HSC were found at the inner border towards the central zones (arrowheads in the enlargement in Fig. 2c4). This indicates that SMMHC-tdTomato-expressing cells are a subgroup of portal vein-associated HSC found primarily in zone 1 of the hepatic lobule. We will therefore use the term 'zone 1-HSC' for this HSC population.

### Lineage tracing of zone 1-HSC

**Figure 3:**
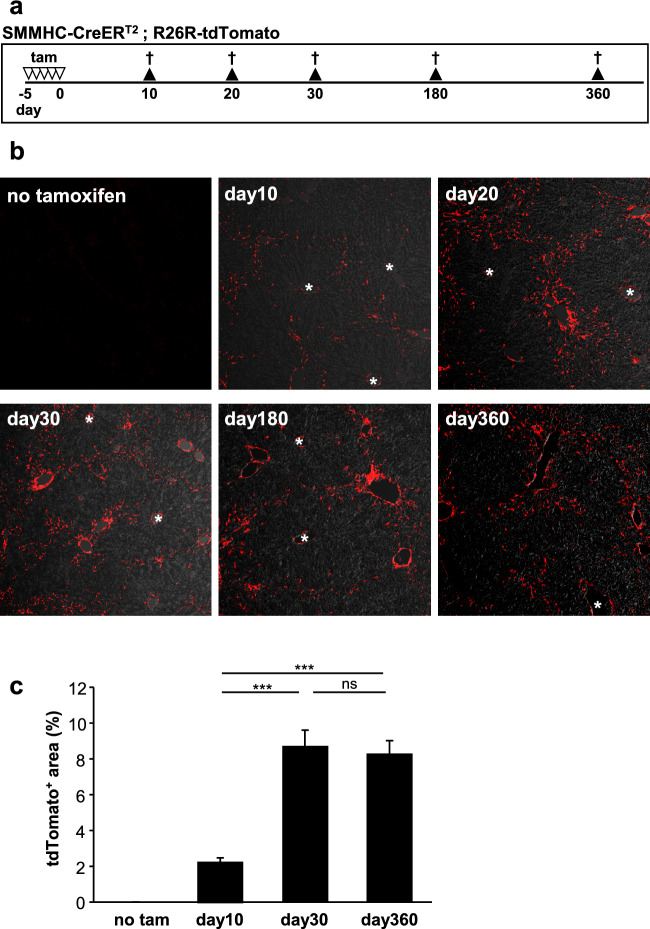
Lineage tracing of zone 1-HSC. (**a**) SMMHC-tdTomato reporter mice were injected with tamoxifen (tam) on 5 consecutive days to induce the expression of the fluorescent dye tdTomato under control of the SMMHC promoter (SMMHC-tdTomato); controls did not receive tamoxifen. Mice were sacrificed at the indicated days after the last tamoxifen injection. Livers were then isolated, fixed and analyzed for tdTomato fluorescence. (**b**) Number of tdTomato-positive zone 1-HSC (red) become visible at around day10 and increase in number until day30. Note that zone 1-HSC remain in the portal area and do rarely cross into central zones even up to 1 year after tamoxifen induction. Bright field is used to show tissue structure. (**c**) Quantitative analysis of tdTomato-positive area without tamoxifen and tamoxifen at day10, day30 and day360. Data are expressed as mean ± SEM (ns, not significant; *** p < 0.0001). Asterisks indicate central veins.

We then performed lineage tracing of SMMHC-tdTomato-positive cells according to the scheme shown in Fig. 3a. Representative images of tdTomato-positive HSC at the indicated timepoints are shown in Fig. 3b (control did not receive tamoxifen). tdTomato expression became manifest approx. 10 days after tamoxifen injection (day10). To our surprise, the SMMHC-tdTomato-labeled HSC remained in zone 1 even 1 year after tamoxifen induction and were not detected in more central areas of the lobule. Quantitative analysis indicates an increase in tdTomato-labeled HSC up to day30 (Fig. 3c). These data show that zone 1-HSC do not occupy the central zones after up to 360 days in a healthy liver.

### SMMHC expression in murine liver

**Figure 4:**
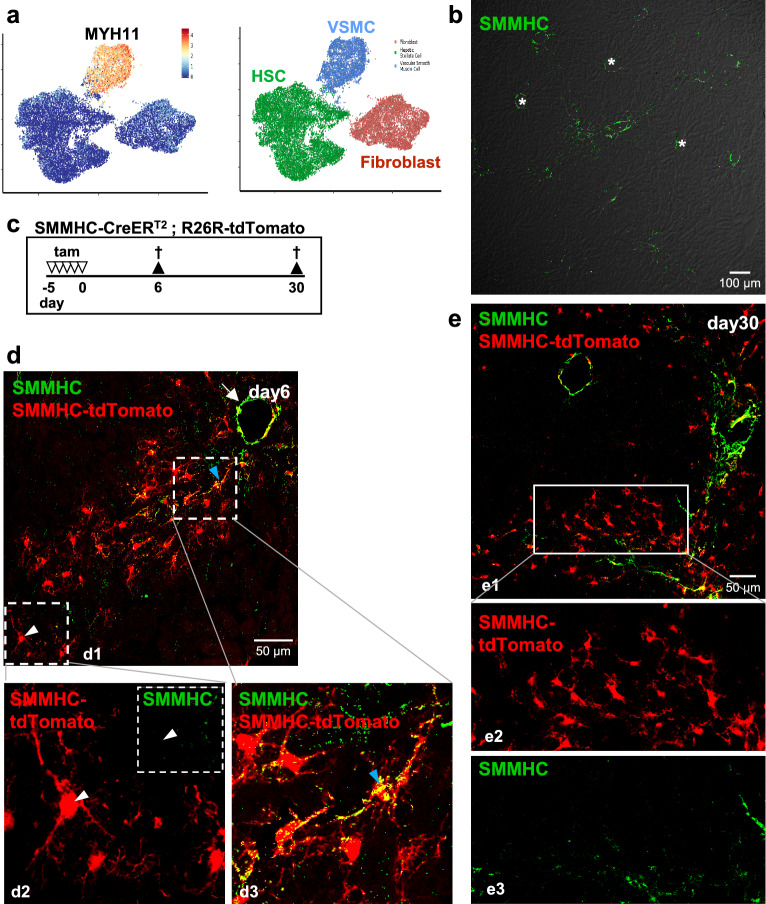
SMMHC expression in murine liver. (**a**) Expression of *Myh11* (SMMHC) mRNA expression in mesenchymal cells of the murine liver. Data from https://shiny.igc.ed.ac.uk/livermesenchyme/^9^. HSC, hepatic stellate cell; VSMC, vascular smooth muscle cell. (**b**) Staining of liver tissue with an antibody against SMMHC (green). Asterisks indicate central veins. (**c**) SMMHC-tdTomato reporter mice were injected with tamoxifen (tam) on 5 consecutive days to induce the expression of the fluorescent dye tdTomato under control of the SMMHC promoter (SMMHC-tdTomato). 6 or 30 days later, mice were sacrificed, and livers were then isolated, fixed and analyzed. tdTomato fluorescence is shown in red. (**d**) SMMHC (green) and tdTomato expression in liver tissue from day6 animals. Apart from vascular SMC (arrow), SMMHC-positive (d3) and SMMHC-negative (d2) (tdTomato-positive) HSC were observed either close to or more distant from the portal area, respectively. (**e**) At day30, only very Livers from day6 animals were stained with antibodies against SMMHC (green). The enlargement (d4) shows a SMMHC-negative but tdTomato-positive zone 1-HSC (white arrowhead). The enlargement d5 shows several SMMHC- and tdTomato-positive cells near the portal field (blue arrowhead).

Analysis of a single cell transcriptomic database^9^ showed strong *Myh11* RNA expression (the gene for SMMHC) in SMC but only very weak RNA signals in HSC (Fig. 4A). This was surprising as tdTomato expression is dependent on an active SMMHC promoter, and HSC in general are not known to derive from SMC. We therefore used immunofluorescence (Fig. 4b) and found SMMHC to be present in vascular SMC and in parenchymal cells of the portal area.

We then looked at possible co-expression of tdTomato and SMMHC 6 and 30 days after tamoxifen (day6, day30; Fig. 4c–e). At day6, most of the tomato-positive cells near the portal vessels were SMMHC-immunopositive whereas those in more distance lacked SMMHC protein (Fig. 4d1; see also the two enlargements for SMMHC-positive (d3; blue arrowhead) and SMMHC-negative (d2; white arrowhead) zone 1-HSC). SMMHC immunolabeling at day30 was strong in SMC of portal and central vessels but hardly recognizable in the parenchyme of the lobule (Fig. 4e). Close-up images reveal that the cells distant from the portal vessel are SMMHC-negative but tdTomato-positive (Fig. 4e2–3). Since we rule out leakage of the SMMHC promoter (see Fig. 3b; no tamoxifen), we conclude that the SMMHC promoter is active only very briefly leading to short time expression of SMMHC protein, but, based on the irreversibility of tdTomato induction, to permanent lineage labeling of zone 1-HSC. Accordingly, since the SMMHC promoter is turned on in only few HSC for a very short time, RNA expression must be low in these cells and, thus, our data are not in contrast to RNAseq data by Dobie et al.^9^.

### Zone 1-HSC cells do not transform into myofibroblasts after CCl_4_ treatment

**Figure 5:**
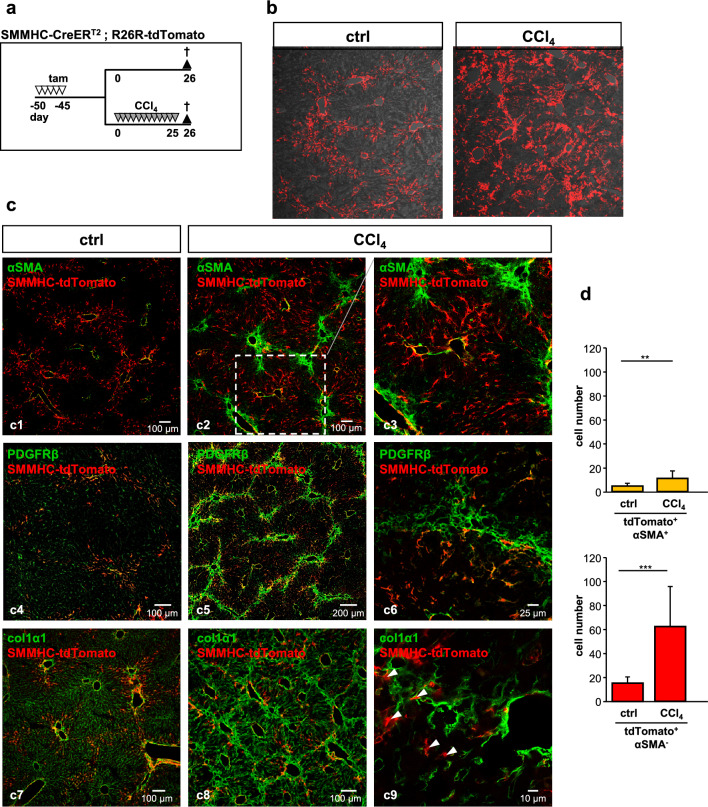
Zone 1-HSC cells do not transform to myofibroblasts after CCl_4_ treatment. (**a**) SMMHC-tdTomato reporter mice were injected with tamoxifen (tam) on 5 consecutive days to induce the expression of the fluorescent dye tdTomato under control of the SMMHC promoter (SMMHC-tdTomato). 45 days later, CCl_4_ was administered (12 injections in 25 days); controls did not receive CCl_4_. Animals were sacrificed one day after the last CCl_4_ injection and livers were then isolated, fixed and analyzed. tdTomato fluorescence is shown in red. (**b**) Loss of zonation of zone 1-HSC in fibrotic liver. (**c**) Antibodies against αSMA (c1-c3, green), PDGFRβ (c4-c6, green) and col1α1 (c7-c9, green) were used. αSMA detects SMC (c1) in control liver and myofibroblasts (c2 and c3) after CCl_4_ treatment. Under fibrotic conditions, migration of zone 1-HSC into the center of the lobules is observed. Enlargement (c3) shows no co-localization of tdTomato and αSMA (except for SMC) indicating that zone 1-HSC do not transform into myofibroblasts. c5 and enlargement in c6 show that myofibroblasts in the portal area are PDGFRβ-positive but negative for tdTomato. In line with this, tdTomato-positive zone 1-HSC were not positive for col1α1 (c8 and c9, arrowheads). (**d**) FACS analysis for tdTomato and αSMA of control and CCl_4_-treated mice. Data are expressed as mean ± SEM (**p < 0.001; ***p < 0.0001).

HSC have been accepted as myofibroblast progenitors during the fibrotic process^4^. To analyze zone 1-HSC behavior during the fibrotic reaction, we treated mice with CCl_4_ according to a conventional scheme (Fig. 5a). CCl_4_ treatment over 4 weeks (3 × per week) lead to distinct fibrosis as indicated by increased collagen expression (PSR stain and col1α1 immunofluorescence) and increased detection of mesenchymal/fibrotic markers such as PDGFRβ, vimentin, αSMA and TGFβ (Supplementary Fig. 2). To our surprise, zone 1-HSC lost their zonation after fibrosis induction as tomato-positive cells were now found throughout the entire lobule (Fig. 5b). Bridging fibrosis and myofibroblasts were identified after CCl_4_ treatment by αSMA, PDGFRβ and col1α1 staining (Fig. 5c2/3, c5/6 and c8/9, respectively; myofibroblasts were αSMA- and PDGFRβ-positive, whereas zone 1-HSC were PDGFRβ-positive but negative for αSMA; Supplementary Fig. 3); myofibroblasts were negative for tdTomato indicating that zone 1-HSC did not contribute to the pool of myofibroblasts after conventional CCl_4_-induced injury.

FACS analysis (Fig. 5d) showed a minor increase in tdTomato/αSMA-double positive cells after CCl_4_ treatment which is likely caused by proliferation of vascular SMC; a stronger increase was seen for tdTomato^+^/αSMA^-^ cells after CCl_4_ treatment which represent the increased number of zone 1-HSC not being myofibroblasts (Fig. 5b,c and Supplementary Fig. 4).

**Figure 6:**
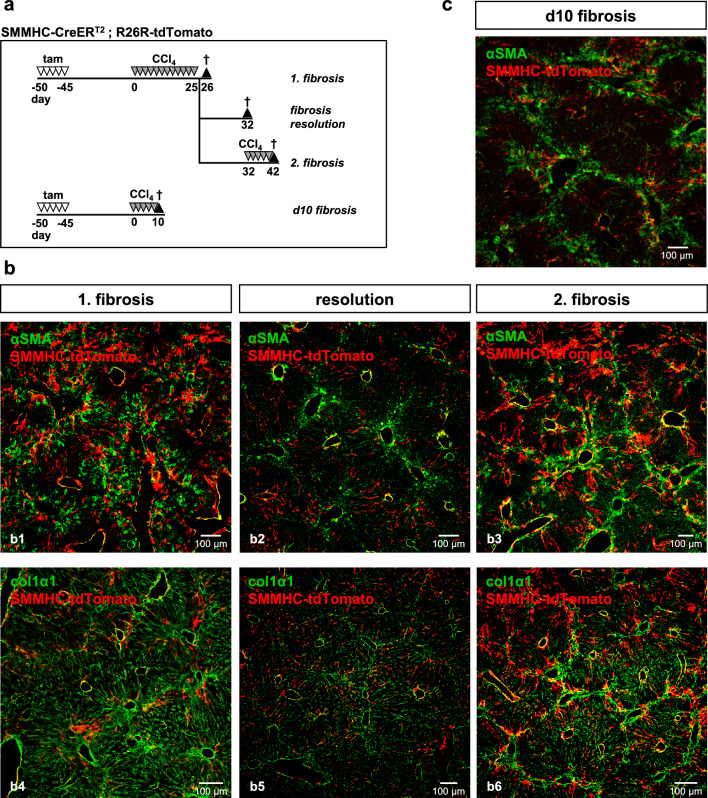
Zone 1-HSC cells do not transform to myofibroblasts after CCl_4_ treatment. (**a**) SMMHC-tdTomato reporter mice were injected with tamoxifen (tam) on 5 consecutive days to induce the expression of the fluorescent dye tdTomato under control of the SMMHC promoter (SMMHC-tdTomato). 45 days later, CCl_4_ was administered (12 injections in 25 days) (*1. fibrosis*) followed by a recovery phase of 1 week (*resolution*) and then followed by another round of 5 CCl_4_ injections (*2. fibrosis*). For short term fibrotic reaction, animals received 5 injections of CCl_4_. Animals were sacrificed one day after the last CCl_4_ injection or after the recovery phase, respectively, and livers were then isolated, fixed and analyzed. tdTomato fluorescence is shown in red. (**b**) No co-localization of tdTomato and was detected under these conditions using antibodies against αSMA (b1-b3, green) and col1α1 (b4-b6, green). (**c**) tdTomato-expressing myofibroblasts were also not detected after the short CCl_4_ protocol.

Since the zonation of zone 1-HSC was lost after CCl_4_ treatment we next investigated a possible myofibroblast fate after a second injury. As shown in Fig. 6a, we treated mice with CCl_4_ for 4 weeks (*1. fibrosis*) followed by a recovery phase of 1 week (*resolution*) and then followed by another round of 5 CCl_4_ injections (*2. fibrosis*). As already shown in Fig. 5, the first CCl_4_ injury led to bridging fibrosis (indicated by αSMA expression) with loss of zonation of zone 1-HSC (Fig. 6b1). During the 7-day resolution phase, myofibroblasts (i.e., αSMA labeling) strongly decreased but tdTomato staining remained in all zones (Fig. 6b2). A second CCl_4_ injury again increased myofibroblasts numbers but as seen in the 1. fibrosis, zone 1-HSC-derived tdTomato-expressing cells did not participate (Fig. 6b3).

To rule out a possible myofibroblast transition at earlier time points during injury, we also tested a short protocol with only 5 CCl_4_ injections and sacrifice after 10 days (Fig. 6a). Even at this early time point, αSMA-positive myofibroblasts were visible, however, we did not detect any co-expression of tdTomato and αSMA (Fig. 6c). We therefore conclude that in the CCl_4_ fibrosis model, zone 1-HSC do not transform into αSMA-positive myofibroblasts.

### Zone 1-HSC cells do not transform to myofibroblasts in a model of MASH

**Figure 7:**
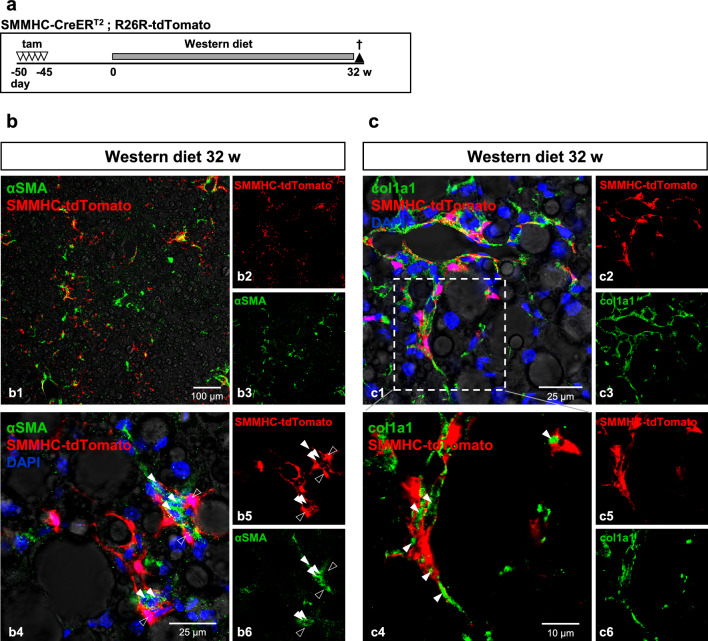
Zone 1-HSC cells do not transform into myofibroblasts in a model of MASH. (**a**) SMMHC-tdTomato reporter mice were injected with tamoxifen (tam) on 5 consecutive days to induce the expression of the fluorescent dye tdTomato under control of the SMMHC promoter (SMMHC-tdTomato). 45 days later, mice received a Western diet consisting of high fat diet supplemented with 0.2% cholesterol and 42 g/l fructose in drinking water. Animals were sacrificed after 32 weeks, and livers were then isolated, fixed and analyzed. tdTomato fluorescence is shown in red. (**b**) Co-localization of tdTomato and αSMA (b1-b3, green; enlargement in b4-b6) was not detected under these conditions. (**c**) Similarly, there was no co-localization of tdTomato and col1α1 (c1-c3, enlargement in c4-c6). Filled arrowheads indicate αSMA^+^ (**b**) or col1α1^+^ (**c**) cells negative for tdTomato; empty arrowheads in (**b**) indicate adjacent αSMA^-^/tdTomato^+^ cells. Note that green staining is in most cases located in the gaps between tdTomato-positive HSC.

We next used a model for metabolic dysfunction-associated steatohepatitis (MASH) to further investigate the role of zone 1-HSC. Mice were first tamoxifen-induced and then received Western diet (i.e., a high fat diet supplemented with cholesterol and fructose) for a maximum of 32 weeks (Fig. 7a; Supplementary Fig. 5a). HE and PSR staining clearly show MASH features namely steatosis, steatohepatitis and chicken wire fibrosis (Supplementary Fig. 5b). αSMA and tdTomato did not co-localize which does not point to the involvement of zone 1-HSC in the development of myofibroblasts (Fig. 7b and c); much rather, in this Western diet model, tdTomato-positive cells appeared to group around αSMA-expressing myofibroblasts (enlargement in Fig. 7b4). Similarly, tdTomato-positive zone 1-HSC were negative for col1α1 but were closely associated with col1α1-expressing myofibroblasts (Fig. 7c). These data further underline that zone 1-HSC are not direct precursors of αSMA/col1α1-producing myofibroblasts.

### Zone 1-HSC participate in sinusoidal capillarization after CCl_4_ treatment

**Figure 8:**
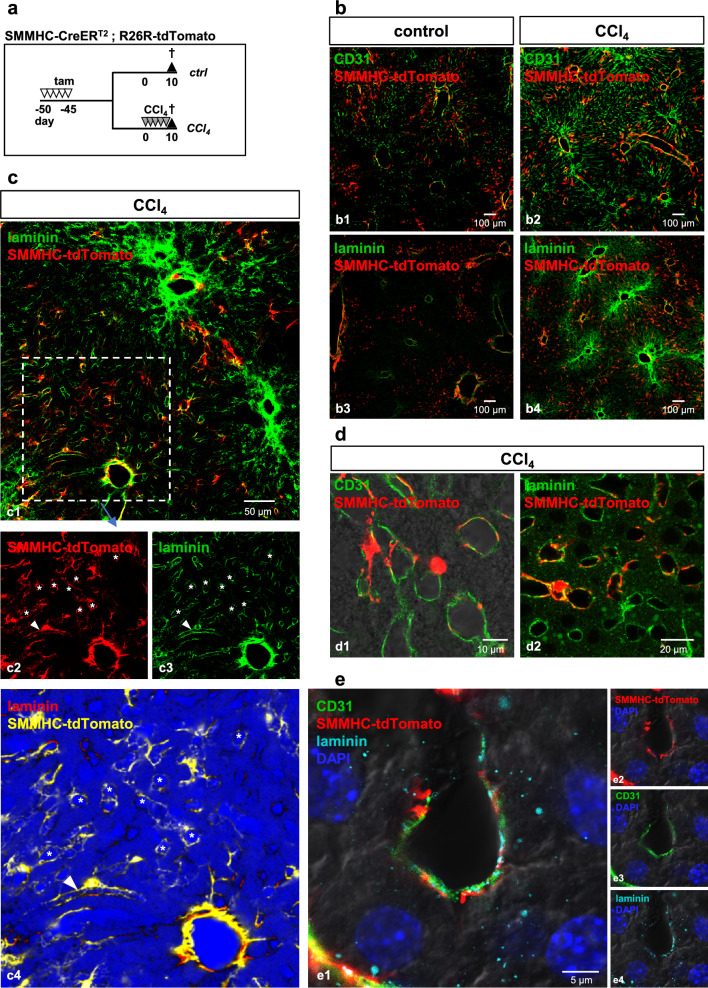
Zone 1-HSC cells participate in sinusoid capillarization after CCl_4_ treatment. (**a**) SMMHC-tdTomato reporter mice were injected with tamoxifen (tam) on 5 consecutive days to induce the expression of the fluorescent dye tdTomato under control of the SMMHC promoter (SMMHC-tdTomato). 45 days later, animals received 5 injections of CCl_4_; controls did not receive CCl_4_. Animals were sacrificed, and livers were then isolated, fixed and analyzed. (**b**) Staining for CD31 and laminin (both green) revealed expression restricted to large vessels in control livers (b1 and b3) and strongly increased expression after fibrosis induction (b2 and b4). (**c**) tdTomato-expression (red, except for c4) was detected in laminin (green)-enriched sinusoidal/tubelike structures (asterisks/arrowhead, respectively). To more clearly show tissue structure (i.e., sinusoidal lumina), laminin is indicated by a gradient from blue to red and tdTomato is shown in yellow in c4. (**d**) Magnification of tdTomato/CD31 (d1) and tdTomato/laminin (d2) co-staining shows close association in hepatic sinusoids. (**e**) Triple staining of a single sinusoid for tdTomato, CD31 and laminin shows close association of endothelial cells, basal lamina and zone-1 HSC. Surrounding hepatocytes can be identified by DAPI-stained nuclei. Single channels are shown in e2-4.

In the healthy liver, sinusoids are lined by fenestrated liver sinusoidal endothelial cells (LSEC). Sinusoids lack basement membrane components (e.g., laminin), CD31 expression and pericyte coverage usually found in capillaries.^18,19^ Capillarization of LSEC may precede the onset of fibrosis and is characterized by the loss of fenestration and synthesis of a basement membrane^20,21^. In addition, capillarization is permissive for HSC activation and myofibroblast formation^22^. To investigate the participation of zone 1-HSC in capillarization, mice were treated with tamoxifen to induce tdTomato expression followed by short-term (5x) CCl_4_ injection (day10; Fig. 8a). Livers were then stained for laminin, a major basement membrane protein, as well as CD31, which has been used as a marker of capillarization^23^. Expression of both laminin and CD31 increased dramatically in the early phase of the fibrotic response (Fig. 8b). At day10, zonation of zone 1-HSC was already disbanded, and tdTomato-positive cells were found close to but not in the fibrotic scar (Fig. 8b2 and b4). tdTomato-expressing HSC were found in close association with laminin in the central zones of the lobule (Fig. 8c). In fact, we identified many sinusoids that were surrounded by tdTomato-positive cells in the fibrotic liver (roundish and tube-like structures marked by asterisks and arrowhead, respectively; Fig. 8c2-c4). Closer examination revealed that CD31 and laminin expression was found around many sinusoids whereas only some sinusoids also showed tdTomato fluorescence (Fig. 8d). Therefore, LSEC capillarization appears to precede zone 1-HSC interaction. Zone 1-HSC-derived tdTomato-expressing cells very closely associated with laminin/CD31 expressing-cells and seemed to wrap around individual sinusoids (Fig. 8d and e). These features are reminiscent of capillary pericytes.

Taken together, our data show that zone 1-HSC do not transform into myofibroblasts during CCl_4_-induced liver fibrosis but rather migrate into central zones to participate in sinusoidal capillarization thereby adopting the typical histology of capillary pericytes.

### Discussion

Heterogeneity of HSC has become evident within the recent years, both under physiological and pathophysiological conditions. Using SMMHC-Cre-based lineage tracing, we have identified a subtype of HSC in the portal area of the liver lobule which we termed zone 1-HSC. Zone 1-HSC appear to be closely related to the PaHSC described by Dobie et al.^9^. Both are located near the portal field and can be identified by high NGFR expression. Are these the same cells? We think that zone 1-HSC are a subgroup of the PaHSC. This interpretation is based on the fact that most but not all tdTomato-labeled HSC show NGFR immunosignals. At day30 after tamoxifen induction, tomato^-^/NGFR^+^ cells can be identified at the inner rim of the zone that is populated by zone 1-HSC (see Fig. 2c). However, lineage tracing over 180 d (Supplementary Fig. 6) revealed that, even after this long period, there is still a rim of tdTomato^-^/NGFR^+^ HSC present. We therefore believe that zone 1-HSC are slightly different from PaHSC. However, we cannot rule out the possibility, that CaHSC (expressing rather low NGFR levels) acquire higher NGFR expression thereby transforming into the innermost tdTomato^-^/NGFR^hi^ cells.

Our data indicate that zone 1-HSC lose zonation by migrating towards the center of the hepatic lobule after fibrosis induction. This behavior contrasts with 'regular' HSC that accumulate periportally by transforming into αSMA-positive myofibroblasts (see graphical abstract). Our data clearly show that zone 1-HSC do not transform into myofibroblasts. What could be the function of zone 1-HSC after migration into central zones? After liver injury, sinusoidal capillarization occurs which usually precedes the onset of fibrosis: Laminin, a major component of capillary basal lamina normally not detectable in hepatic sinusoids, is heavily deposited in the space of Disse^19,24^ thus, in combination with collagen IV, a perisinusoidal basement membrane is formed. The sinusoidal endothelium becomes defenestrated and transforms into a vascular type endothelium which is e.g. characterized by increased CD31 expression. Thus, laminin and CD31 are established markers of capillarization^20,25,26^. In our experiments, both laminin and CD31 were strongly increased after CCl_4_ treatment. Formation of closed laminin-associated endothelium encircling hepatic sinusoids is reminiscent of the structure of capillaries. Together with tomato-positive zone 1-HSC, ring- and tube-like structures were obvious after injury (Fig. 8c,d) which even show laminin immunosignals sandwiched between CD31 and tomato staining (Fig. 8e1). Thus, zone 1-HSC appear to participate in the process of sinusoidal capillarization by adopting a classic pericyte phenotype after fibrosis induction. We have not investigated the function of these newly formed pericytes, but prevention of free perfusion of the otherwise relatively open sinusoidal space with possible injury-related toxins appears plausible.

There are of course some limitations of the study. Our data would be corroborated by a marker that discriminates HSC, SMC and other mesenchymal cells such as mesenchymal stromal cells/stem cells. Our study uses two different fibrosis models (toxic and dietary). We selected these two models since they are frequently used, easy to assess and (in the case of the MASH model) reflect the phenotype in humans. Since zone 1-HSC can be identified and characterised in both models, our choice appears to be valid; however, other fibrosis models will be needed to further explore this HSC subtype. In addition, zone 1-HSC and their functions need to be addressed in other animal systems as well as in human liver.

In summary, we here describe a novel subtype of HSC. The main features of these zone 1-HSC are specific zonation under physiological conditions, loss of zonation after injury and adaption of a capillary pericyte rather than a myofibroblast phenotype. Of course, the elucidation of the exact function of zone 1-HSC and the interaction with 'regular', more central HSC subtypes is mandatory for the future.

## Materials and Methods

All authors had access to the study data and had reviewed and approved the final manuscript.

### Animals

All animal experiments in this study conform to the Animal Research: Reporting of In Vivo Experiments (ARRIVE) guidelines (http://www.nc3rs.org.uk/arrive-guidelines). The animal procedures were performed according to the guidelines from directive 2010/63/EU of the European Parliament on the protection of animals used for scientific purposes. All experiments were approved by the local animal care committee (Bezirksregierung Unterfranken, Az 55.2-2532-2-502 and 2-1471). Euthanasia was performed by cervical dislocation under isoflurane anesthesia. All mice were bred in our facility.

Mice were housed in standard mouse cages (267x207x140 mm; maximally three animals/cage) with woodchip bedding material and under conventional laboratory conditions (constant room temperature (22 °C), humidity level (55%), a 12-h light/12-h dark cycle (lights on at 6 am). Standard rodent diet (Altromin, Lage, Germany) and water were available ad libitum.

### Generation and genotyping of SMMHC-tdTomato reporter mice

For lineage tracing studies, SMMHC-tdTomato reporter mice expressing the fluorescent dye tdTomato under control of the SMMHC promoter were obtained by crossing SMMHC-CreER^T2^ mice (JAX #019079; genetic background: C57Bl6) with a tdTomato reporter line (Ai14; JAX #007914; genetic background: C57Bl6). As the SMMHC-CreER^T2^ gene is located on the y-chromosome, only male offspring express Cre recombinase; thus, only male mice (total of 126) were used in this study. Genotyping was performed by PCR analysis (see Supplementary data Table 1 for list of primers).

### Tamoxifen injection

Male SMMHC-tdTomato reporter mice aged 6–8 weeks were injected with tamoxifen (dissolved in Miglyol 812; 1 mg i.p.) on five consecutive days.

### CCl_4_ administration

CCl_4_ (1 µl/g body weight) was injected intraperitonially three times a week for 4 weeks to induce primary parenchymal liver fibrosis. Fibrosis reversal was analyzed after pausing for one week after the last CCl_4_ injection (i.e., 4 weeks treatment, 1 week cessation). For a second fibrosis induction, animals with 4 weeks CCl_4_ paused for 1 week and then received 3 additional CCl_4_ injections within 1 week. Moreover, early fibrosis injury was examined after 4 injections of CCl_4_ (within 9 days, sacrifice on day 10). At the end of last CCl_4_ treatment, animals were euthanized and livers were perfused with sterile PBS followed by 2% paraformaldehyde (PFA). Livers were incubated with 2% PFA at 4 °C for 2 h. The liver tissues were then incubated overnight at 4 °C in 20% sucrose and embedded in the tissue-Tek medium for later use.

### Induction of metabolic dysfunction-associated steatohepatitis (MASH)

SMMHC-tdTomato reporter mice were fed Western diet (21% fat, 0.2% cholesterol) and 42 g/l fructose in drinking water for up to 32 weeks. After euthanasia, livers were perfused with sterile PBS followed by 2% PFA) Livers were incubated with 2% PFA at 4 °C for 2 h. The liver tissues were then incubated overnight at 4 °C in 20% sucrose and embedded in the tissue-Tek medium for later use.

### Immunohistochemical analysis

Cryosections of fixed liver preparations were cut, permeabilized and incubated overnight at 4 °C either alone or with primary antibodies as listed in the Supplementary data Table 2. Positive controls for the antibodies against CD31, PDGFRβ, αSMA and col1A1 in lung tissue are shown in Supplementary Fig. 7. Secondary antibodies were incubated in antibody diluent either alone or in combination with DAPI (1:1000; Applichem, Heidelberg, Germany) for one hour at RT. The sections were mounted in Mowiol and were evaluated using a confocal microscope (Leica TCS SP8). Differential interference contrast polarizing filter (DIC/pol) was used for visualization of tissue structure.

### Hematoxylin and eosin (H&E) and Sirius Red morphometry

Dewaxed, hydrated liver tissue sections were incubated in hematoxylin (MORPHISTO, Frankfurt, Germany) for 10 min, washed in tap water for 15 min followed by incubation in eosin (MORPHISTO, Frankfurt, Germany) for 2 min and rinsed in distilled water. Stained tissue sections were dehydrated in increasing concentrations of ethanol and finally immersed in xylene and mounted. For collagen staining, tissue sections were incubated in Sirius Red solution (MORPHISTO, Frankfurt, Germany) for 1 h, washed two times in acidified water (0.5% acetic acid) followed by dehydration; tissues were then immersed in xylene and mounted. H&E- and Sirius Red-stained sections were visualized via Keyence Microscope (Modellreihe BZ-X) with appropriate filters.

### Analysis of tdTomato-positive cells via flow cytometry

After homogenization of liver tissues and subsequent incubation with 0.4% collagenase IV (Sigma, Tauffenkirchen, Germany),1.6 nM DNaseI (Applichem, Munich, Germany) in 154 mM NaCl, 5.6 mM KCl, 5.5 mM glucose, 20.1 mM HEPES, 25 mM NaHCO_3_, 2 mM CaCl_2_, 2 mM MgCl_2_, pH 7.4, for 30 min at 37 °C, the homogenate was filtered (via 100 μm cell strainer (BD Bioscience, Leipzig, Germany) and centrifuged at 21 × *g* for 4 min to remove hepatocytes. The supernatant was incubated with RBC solution for 10 min at RT and centrifuged at 300 × *g*. The pellet was washed, and cells were counted. One million cells were used for FACS staining and incubated with Alexa flour 488 αSMA and Dye eflour 780 Fixable viability dye. Data acquisition was done on a Attune NxT Flow Cytometer (ThermoFischer, Darmstadt, Germany) and analyzed via FlowJo software.

### Statistics

Data are expressed as mean ± SEM. For calculation of statistical tests, GraphPadPrism 9.0 for Windows was used. Two independent groups were compared by unpaired, two-sided T-test. One-way ANOVA followed by Tukey post-hoc test was used to compare multiple groups of one genotype.

### Supplementary Information

Supplementary Information.

## Data Availability

The datasets generated during and/or analysed during the current study are available from the corresponding author on reasonable request.
