# Supplementary material for: Hepatic stellate cells in zone 1 engage in capillarization rather than myofibroblast formation in murine liver fibrosis
Source: Sci Rep. 2024 Aug 13;14:18840. doi: 10.1038/s41598-024-69898-z (PMC11322391; doi:10.1038/s41598-024-69898-z)
Supplement: Supplementary file 1 — Supplementary Information. [file 41598_2024_69898_MOESM1_ESM.pdf]

Supplementary Data

Hepatic stellate cells in zone 1 engage in capillarization rather than myofibroblast formation in murine liver fibrosis

Muhammad Ashfaq Khan, Julian Fischer, Leon Harrer, Fabian Schwiering, Dieter Groneberg, and Andreas Friebe*

Physiologisches Institut, Julius-Maximilians-Universität Würzburg, 97070 Würzburg, Germany

Table 1: primers used

gene	primer name	sequence
**SMMHC-CreER^{T2}**	SMWT1	TGACCCCATCTCTTCACTCC
	SMWT2	AACTCCACGACCACCTCATC
	phCREAS1	AGTCCCTCACATCCTCAGGTT
**tdTomato**	tomato-545-5'	TCCCACAACGAGGACTACA
	tomato-545-3'	CCCATGGTCTTCTTCTGCATTA
	Rosa26-5'	AAGGGAGCTGCAGTGGAGTA
	Rosa26-3'	CCGAAAATCTGTGGGAAGTC

Table 2: primary antibodies used

antibody	host	dilution	order #	manufacturer
anti-glutamine synthetase	rabbit	1:200	ab16802	Abcam, Cambridge, UK
anti-NGFR/TNFRSF16	goat	1:300	AF1157	R&D systems, Minneapolis, USA
recombinant anti-alpha smooth muscle actin	rabbit	1:500	ab32575	Abcam, Cambridge, UK
anti-collagen I	rabbit	1:500	ab21286	Abcam, Cambridge, UK
anti-CD31	rat	1:200	550274	BD Biosciences, Heidelberg, Germany
anti-F4/80	rat	1:200	MCA497G	Bio-Rad, Feldkirchen, Germany
anti-SMMHC	rat	1:200	ab125884	Abcam, Cambridge, UK
anti-PDGFRβ	goat	1:200	AF1042	R&D Systems, Minneapolis, USA
anti-laminin	rabbit	1:200	BP8037	Sigma-Aldrich, München, Germany
Alexa 488-conjugated IgG	donkey	1:500	A21208	Invitrogen, Darmstadt, Germany
Alexa 555-conjugated IgG	donkey	1:500	A31572	Invitrogen, Darmstadt, Germany
Alexa 633-conjugated IgG	donkey	1:500	A21070	Invitrogen, Darmstadt, Germany
Alexa-647-conjugated IgG	donkey	1:500	A78947	Invitrogen, Darmstadt, Germany
Alexa 488-conjugated anti-alpha smooth muscle actin	mouse	1:200	53976080	Invitrogen, Darmstadt, Germany

RT-qPCR

After euthanasia, livers were perfused with 0.9% sodium chloride in DEPC water. The tissue was immediately snap frozen in liquid nitrogen and stored at -20°C. Using QIAshredder and Qiagen Rneasy Mini Kit, total RNA was isolated from 15 mg lung tissue (right lung). RNA concentration and purity were assessed by a microvolume spectrophotometer (SimpliNano Spectrophotometer, Biochrom). cDNA was synthesized using 50 ng/µl of total RNA according to the High-Capacity cDNA Reverse Transcription Kit by Thermo Fisher Scientific. qPCR was performed with the CFX96 Real-Time PCR Detection System, Bio-Rad, using total cDNA as a template, specific primers for PDGFRβ and TGFβ and the SsoFast EvaGreen Supermix by Bio-Rad. PrimerBlast by NCBI was used to design primers (Table 1) based on the mRNA sequence of Mus musculus. All primers were produced by Sigma-Aldrich, München.

Table 3: qPCR primers used

Gene	Primer	Sequence (5'-3')
**PDGFRβ**	forward	TCCAGGAGTGATACCAGCTTT
	reverse	CAGGAGCCATAACACGGACA
**TGFβ**	forward	CATGGACTGGTGAAACGA
	reverse	CGCGAGCCTTAGTTTGGACA

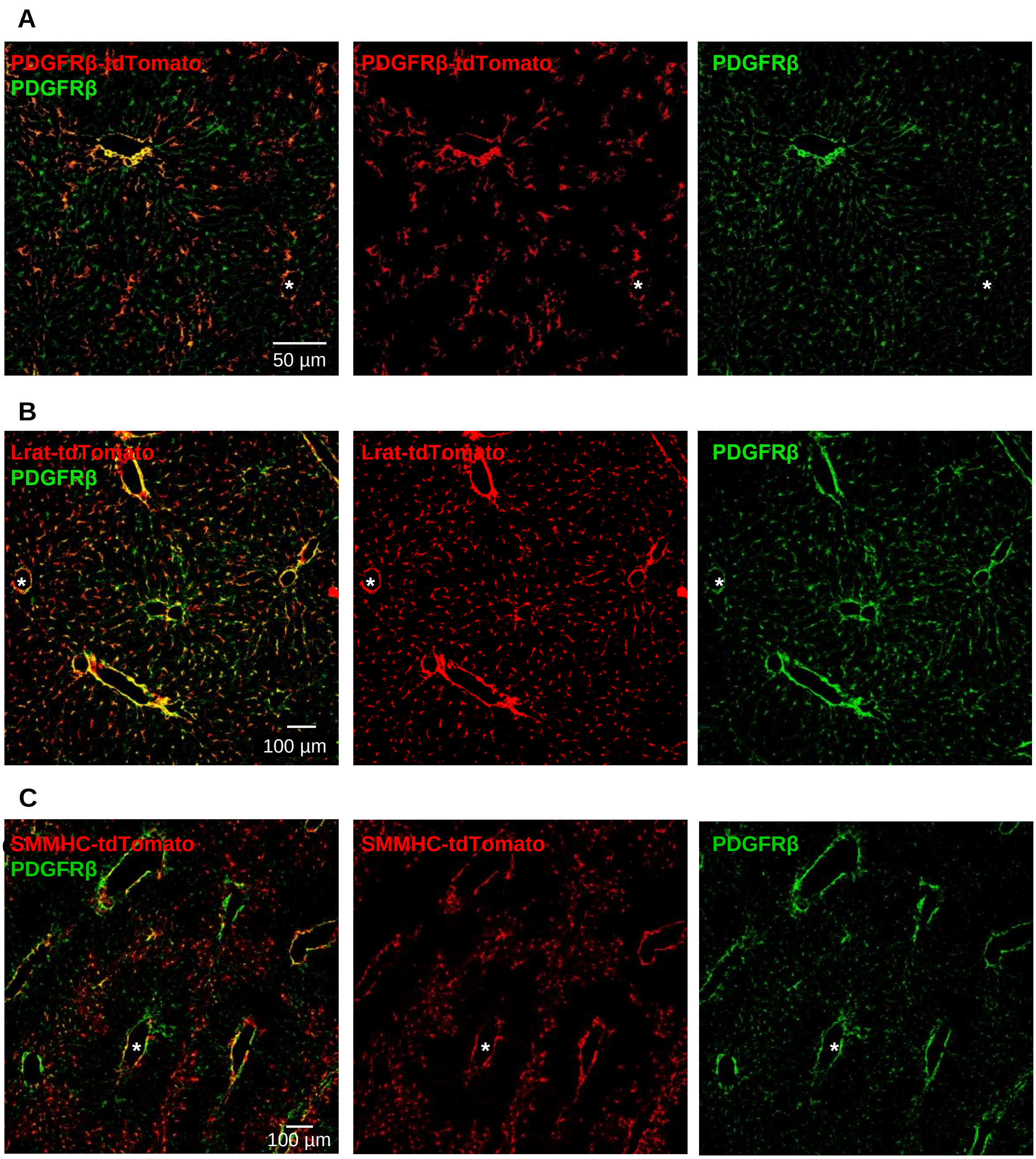

Supplementary Figure 1: tdTomato expression under the control of Lrat-Cre, PDGFRβ-CreER^{T2} and SMMHC-CreER^{T2}

Reporter mice expressing the fluorescent dye tdTomato under control of the Lrat, PDGFRβ or SMMHC promoter were obtained by crossing PDGFRβ-CreER^{T2} (**A**), Lrat-Cre (**B**) and SMMHC-CreER^{T2} (**C**) with a tdTomato reporter line (Ai14; JAX #007914; genetic background: C57Bl6). All three Cre lines are known to target HSC/pericytes. An antibody against PDGFRβ was used as marker for HSC and SMC. The two inducible lines were treated with tamoxifen and livers isolated 30 days later. The Lrat-Cre tomato-labeled all HSC whereas stochastic labeling of HSC was seen under control of the PDGFRβ-CreER^{T2}. Induction of SMMHC-CreER^{T2} led to tomato-labeling of HSC in the periportal region of the hepatic lobule. All three Cre lines labeled hepatic SMC. Asterisks indicate central veins.

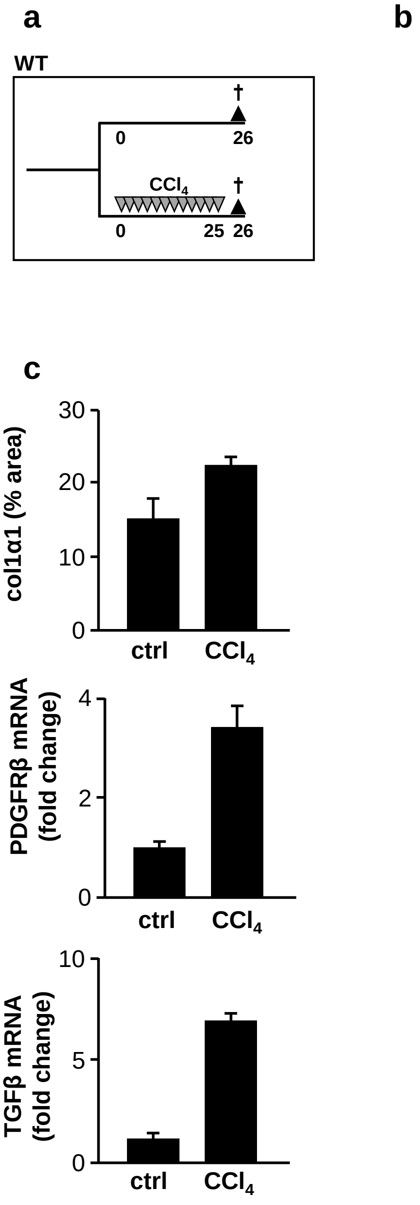

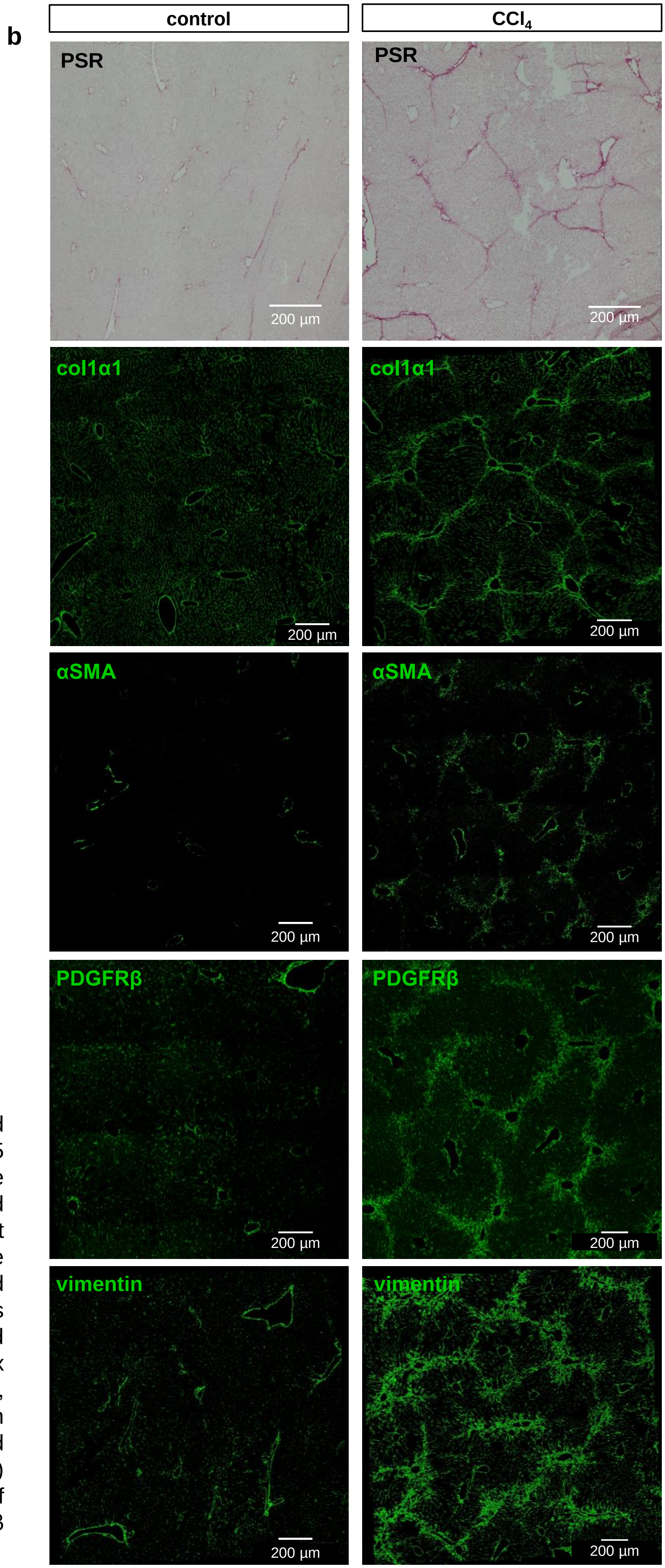

Supplementary Figure 2: Establishment of CCl_4 fibrosis

(**a**) WT mice were injected with CCl_4 (12 injections in 25 days); controls did not receive CCl_4. Animals were sacrificed on d26 (one day after the last CCl_4 injection) and livers were then isolated, fixed and analyzed. (**b**) After fibrosis induction, mice showed increased extracellular matrix deposition (PSR, col1α1), myofibroblast formation (αSMA, PDGFRβ and vimentin) and TGFβ. (**c**) Quantitative analysis of col1α1, PDGFRβ and TGFβ mRNA expression.

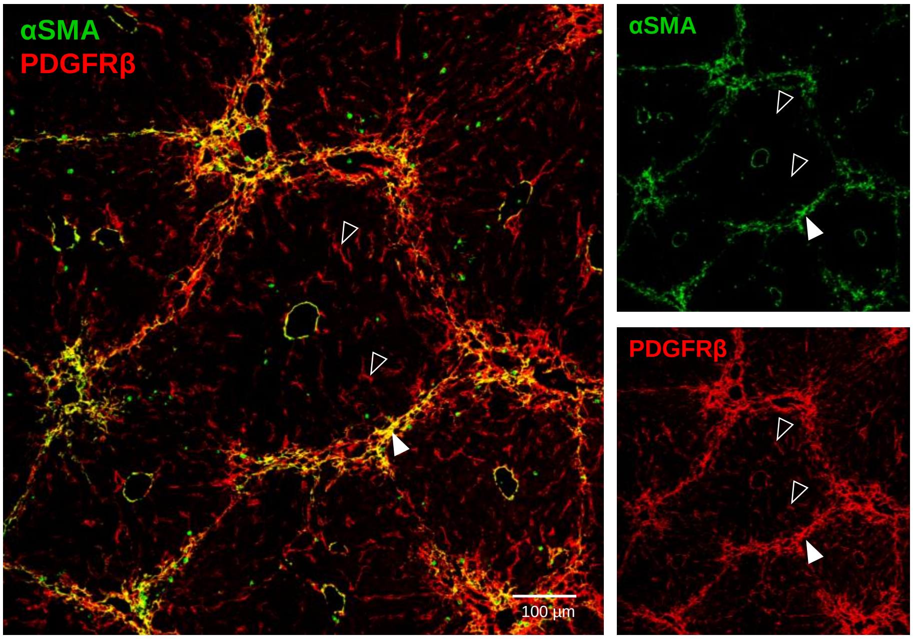

Supplementary Figure 3: Double staining for αSMA and PDGFRβ discerns myofibroblasts from zone 1-HSC.

SMMHC-tdTomato reporter mice were treated as described in Fig. 4a. Double staining with antibodies against αSMA (green) and PDGFRβ (red) showed double positive cells in the fibrotic area (yellow, filled arrowhead) and PDGFRβ-positive, αSMA-negative cells in the central zones of the lobule (red, empty arrowheads). The latter correspond to zone 1-HSC after fibrosis induction whereas the first are myofibroblasts.

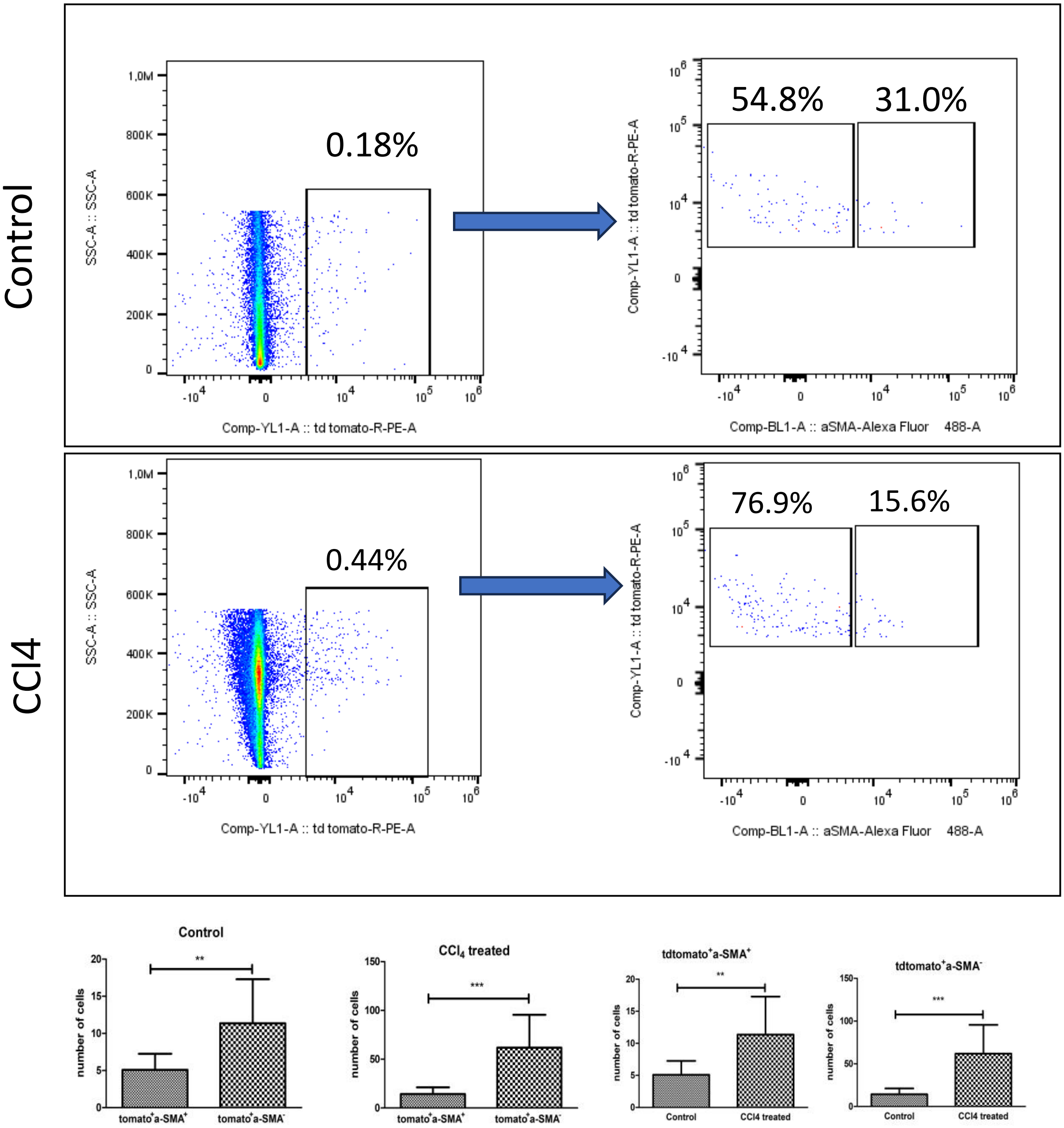

Supplementary Figure 4: Gating strategy for FACS.

After exclusion of debris and dead cells, cells were gated on tdTomato-positive cells and further subdivided into tdTomato⁺/αSMA⁻ and tdTomato⁺/αSMA⁺ cells in control and CCl_4-treated SMMHC-tdTomato reporter mice. Data are expressed as mean ± SEM (** p<0.001; *** p<0.0001).

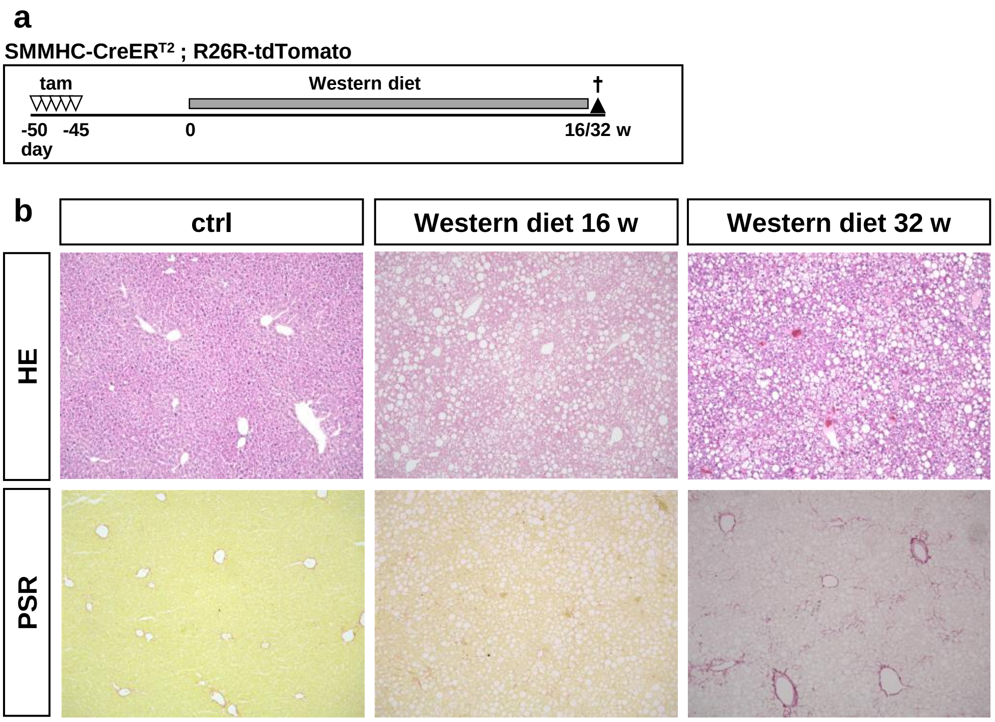

Supplementary Figure 5: Western diet induces mild to moderate fibrosis
(**a**) SMMHC-tdTomato reporter mice were injected with tamoxifen (tam) on 5 consecutive days to induce the expression of the fluorescent dye tdTomato under control of the SMMHC promotor (SMMHC-tdTomato). 45 days later, mice received a Western diet consisting of high fat diet supplemented with 0.2% cholesterol and 40 g/l fructose in drinking water. Animals were sacrificed after 16-32 weeks, and livers were then isolated, fixed and analyzed. (**b**) HE and PSR stainings show mild (16 weeks) and moderate (32 weeks) fat accumulation and collagen deposition as indicator of MASH.

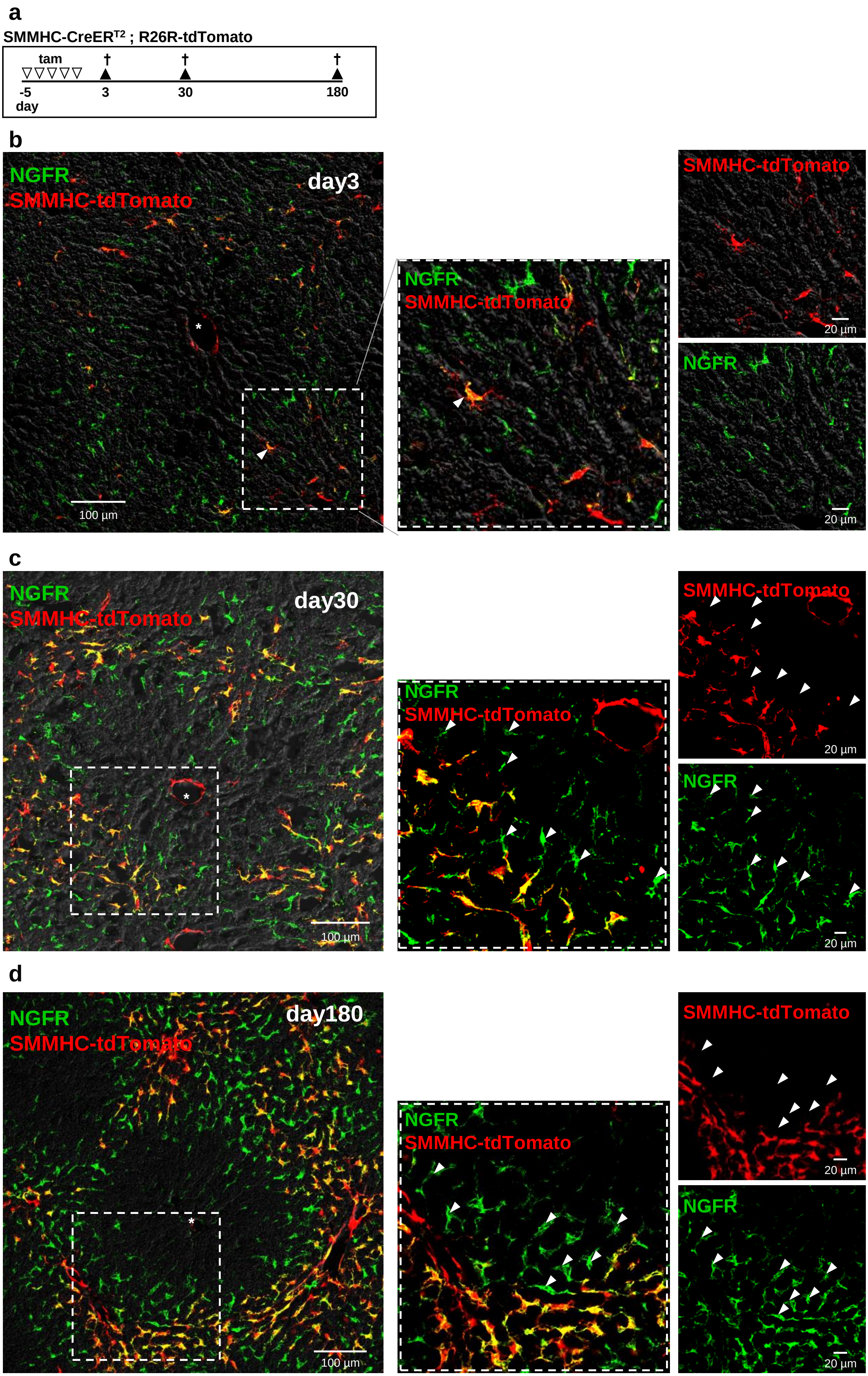
a
SMMHC-CreERT2 ; R26R-tdTomato
tam
-5
day
3
30
180
b
NGFR
SMMHC-tdTomato
day3
100 µm
NGFR
SMMHC-tdTomato
SMMHC-tdTomato
20 µm
NGFR
20 µm
c
NGFR
SMMHC-tdTomato
day30
100 µm
NGFR
SMMHC-tdTomato
SMMHC-tdTomato
20 µm
NGFR
20 µm
d
NGFR
SMMHC-tdTomato
day180
100 µm
NGFR
SMMHC-tdTomato
SMMHC-tdTomato
20 µm
NGFR
20 µm

Supplementary Figure 6: Zone 1-HSC are a subtype of NGFR-positive periportal HSC

(**a**) SMMHC-tdTomato reporter mice were injected with tamoxifen (tam) on 5 consecutive days to induce the expression of the fluorescent dye tdTomato under control of the SMMHC promotor (SMMHC-tdTomato). Mice were sacrificed at day3 (**b**), day30 (**c**) and day180 (**d**) after the last tamoxifen injection. Livers were then isolated, fixed and analyzed for tdTomato fluorescence. At all time points, the tdTomato-positive cells expressed NGFR. However, especially at later time points, NGFR-positive tdTomato-negative cells were present at the central boarder of zone 1. tdTomato-positive zone 1-HSC are not congruent with the NGFR-positive HSC, thus they must be considered as a subtype.

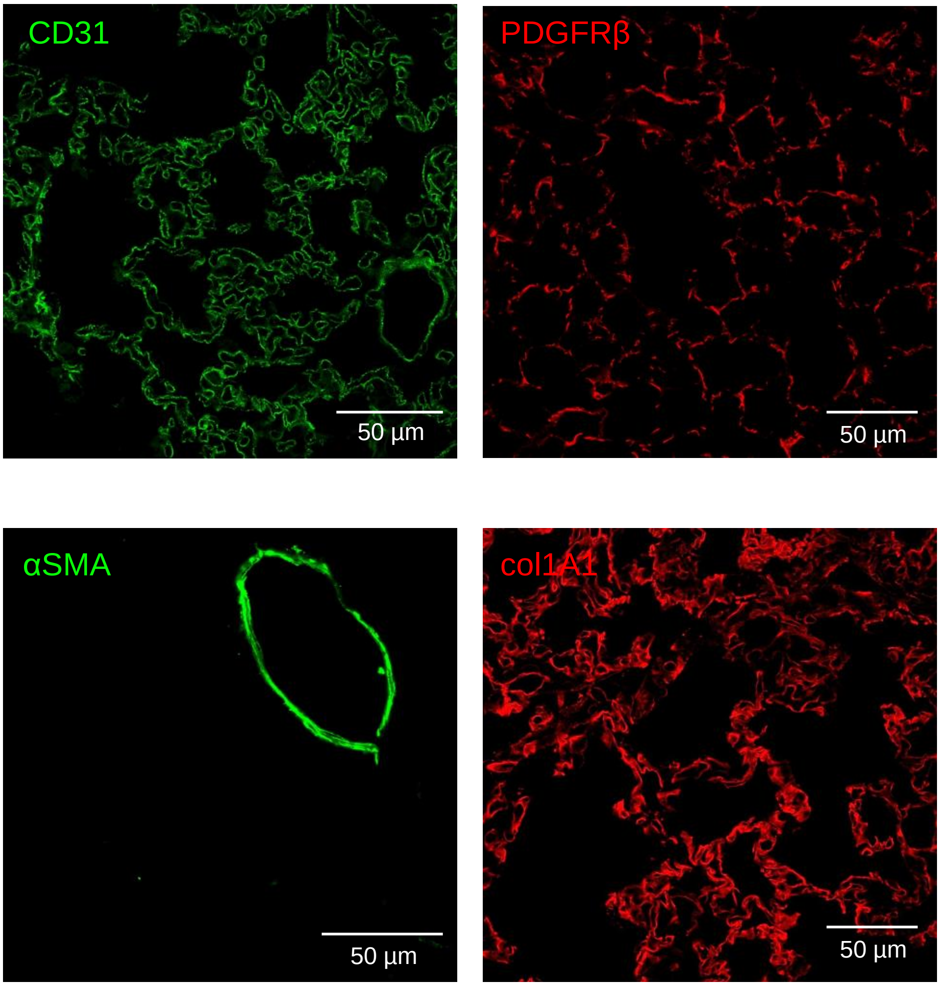

Supplementary Figure 7: Positive controls CD31, PDGFRβ, αSMA and col1A1 in lung tissue.

The antibody against CD31 stained endothelial cells, whereas that against PDGFRβ marked lung pericytes. The antibody against αSMA only stained SMC in pulmonary vessels. Col1A1 stained extracellular matrix within the alveolar wall.
